# Supplementary material for: Peptide barcodes in dogs affected by mitral valve disease with and without pulmonary hypertension using MALDI-TOF MS and LC-MS/MS
Source: PLoS One. 2021 Aug 12;16(8):e0255611. doi: 10.1371/journal.pone.0255611 (PMC8360550; doi:10.1371/journal.pone.0255611)
Supplement: Attachment — Submitted filename: Response to Reviewers_Final 20210627.pdf [file pone.0255611.s001.pdf]

Dear Editors-in-Chief, PLOS ONE,

Thank you very much for giving us an opportunity to revise our work "Peptide barcodes in dogs affected by mitral valve disease with and without pulmonary hypertension using MALDI-TOF MS and LC-MS/MS." After we revised it in response to the journal requirement and reviewers' concerns, we have highlighted changes in different colors. We hope this makes it easy for you to locate where they were updated.

Please see the attached file for the revision, which is highlighted in green for Journal Requirements, yellow for Reviewer 1, blue for Reviewer 2.

Journal Requirement:

Please review your reference list to ensure that it is complete and correct. If you have cited papers that have been retracted, please include the rationale for doing so in the manuscript text, or remove these references and replace them with relevant current references. Any changes to the reference list should be mentioned in the rebuttal letter that accompanies your revised manuscript. If you need to cite a retracted article, indicate the article's retracted status in the References list and also include a citation and full reference for the retraction notice.

We have checked our references and found none of them have been retracted. We deleted reference 11 as it duplicated reference 6 in the previous manuscript (line 519-520 of the track-change file). The revision manuscript then has 58 references relevant the PLOS ONE's requirements.

1. Please ensure that your manuscript meets PLOS ONE's style requirements, including those for file naming.

   Please be confirmed that we have managed the manuscript to meet PLOS ONE's style requirements.

2. We suggest you thoroughly copyedit your manuscript for language usage, spelling, and grammar.

   We have thoroughly revised our manuscript for language usage, spelling, and grammar. Moreover, the majority of this manuscript was edited for English language, grammar, punctuation, spelling, and overall style by the highly qualified native English-speaking editors at SNAS. The certificate is attached.

3. Please upload a copy of Supporting Information File which you refer to in your text (line 442).

   We have no supporting information that would like to be uploaded in this study. We just listed this heading topic following PLOS ONE's manuscript formatting guidelines. We have added 'Not applicable' below this heading (line 678 of the track-change file, and 663 of the non-track-change file).

Reviewer 1:

The authors in this manuscript used mass spectrometry-based proteomics to differentiate between the different stages of mitral valve disease (MVD) found in dogs. Matrix-assisted laser desorption/ionization timeof-flight mass spectrometry (MALDI-TOF MS) and liquid chromatography-tandem mass spectrometry (LC-MS/MS) was used to compare the serum proteomes from healthy dogs, dogs with MVD stage B (MVD B, asymptomatic stage), MVD stage C (MVD C, symptomatic stage), MVD stage B with PH (MVD B PH) and MVD stage C with PH (MVD C PH). This resulted in the identification of the differences in the serum peptide barcodes of dogs in the MVD B, MVD C, MVD B PH, MVD C PH, and normal control groups. Six discriminatory peptides at m/z 1,225.60, 1,363.85, 1,688.71, 1789.52, 2020.21, and 2156.42 were altered in their abundance, representing changes in the protein levels of peptide barcode components among the groups. These results are helpful in the diagnosis of different stages of MVD with PH or without PH (pulmonary hypertension). Overall, I recommend the publication of this work as it demonstrates the application of mass spectrometry-based proteomics in diagnosis.

There is no specific concern from reviewer 1. Thank you very much for your consideration recommending the publication of our study.

Reviewer 2:

1. Of concern is the choice of the 'normal' group, having a median age of about half that of the other groups. Is it possible that some of the barcodes identified could become non-specific to one or more groups if the 'normal' group had an increased age? A discussion point on the choice of the 'normal' group and limitations of this group with age is needed.

   Because mitral valve disease and pulmonary hypertension, the conditions we have focused on in this study, are commonly found in older dogs, usually more than 5 years old. Accordingly, we designed to recruit dogs at least 5 years old to match the age between normal and the disease groups. However, despite this attempt, the median age of the normal group was still a half younger than the other groups. The reason for this situation was the lack of aging dogs without any concurrent systemic diseases. Therefore, most of the enrolled dogs in the normal group were much younger than dogs in the group with mitral valve disease and pulmonary hypertension. For this concern, it is still possible that some peptide barcodes of the normal group may become non-specific to the disease group if the normal group has an increased age. This limitation has been additionally discussed as the study's limitations in the discussion part (line 407-420 of the track-change file, and line 406-420 of the non-track-change file).

2. A practical limitation would include the access to MALDI and LC equipment to a veterinary clinic. It is very unlikely a clinic would purchase such equipment, thus in several instances the analyses of samples would have to be outsourced. Consider a discussion point about this limitation.

Presently, MALDI-TOF MS is being used in human medicine as a common diagnostic test for early diagnosis and monitoring, particularly in the field of neoplastic illnesses. It has been used to detect cancer in its early stages in a variety of cancers. Conversely, in veterinary medicine, there is a limitation in reaching MALDI-TOF and LC mass spectrometry in routine practice due to the high cost of the equipment. However, this study could add a point of view in the proteomic field of veterinary cardiology. Although the use of MALDI-TOF as a diagnostic method in dogs with heart diseases is still currently limited in the present day. In the future, the findings of this study may assist in the development of a novel diagnostic method for dogs with heart diseases. We have already added the discussion of this point as a study limitation (line 420-429 of the track-change file, 420-428 of the non-track-change file).

3. Was pro B-type-natriuretic peptide data available for the animals examined? If so, was it also examined via mass spectrometry, and how did the differing methods compare?

We have no data available of N-terminal B-type-natriuretic peptides for the enrolled animals in this study. To our knowledge, no study reported on comparing the measurement of N-terminal B-type-natriuretic peptide levels and mass spectrometry as diagnostic methods in dogs with mitral valve disease and pulmonary hypertension. This is an interesting topic that recommends further investigation.

4. Minor: though well written, some correction to punctuation is needed through the manuscript.

We thoroughly revised the language usage and punctuation in our manuscript.

Thank you for your consideration.

Sincerely Yours,

Walasinee Sakcamduang DVM, PhD
E-mail: walasinee.sak@mahidol.ac.th
